# Generalized Uniqueness Theorem for Ordinary Differential Equations in Banach Spaces

**DOI:** 10.1155/2014/272479

**Published:** 2014-02-10

**Authors:** Ezzat R. Hassan, M. Sh. Alhuthali, M. M. Al-Ghanmi

**Affiliations:** Department of Mathematics, Faculty of Science, King Abdulaziz University, P.O. Box 80203, Jeddah 21589, Saudi Arabia

## Abstract

We consider nonlinear ordinary differential equations in Banach spaces. Uniqueness criterion for the Cauchy problem is given when any of the standard dissipative-type conditions does apply. A similar scalar result has been studied by Majorana (1991). Useful examples of reflexive Banach spaces whose positive cones have empty interior has been given as well.

## 1. Introduction

Throughout the last century most of the efforts are concentrated on the study of the classical Cauchy problem, also called the initial value problem and denoted by IVP:

(1) $$\begin{array}{c} x^{\prime }=f\left(t,x\right),\;\;x\left(0\right)=0, \end{array}$$

where *f* : [0,1] × *E* → *E* and *E* is a real Banach space. In the finite dimensional case the existence is guaranteed by Peano's theorem. In order to put our results into context, let us start by formulating the classical theorem of Peano.

**Theorem 1 (see [1])**

Let *E* = ℝ^*n*^ and *f* ∈ *C*([0,1] × ℝ^*n*^; ℝ^*n*^). Then (1) has a local solution.

Such an infinite dimensional Cauchy problem may have no solutions. Dieudonné [2] provided the first example of a continuous map from an infinitely dimensional nonreflexive Banach space *C*_0_ for which there is no solution to the related Cauchy problem (1). Many counterexamples in various infinite dimensional reflexive as well as nonreflexive Banach spaces followed, for example, [3–6]. Afterwards, Godunov [7] proved that Theorem 1 is false in every infinite dimensional Banach space. It turned out that continuity alone, of the function *f*, is not sufficient to prove a local existence theorem in the case where *E* is infinite dimensional. In order to obtain suitable extensions for the continuity notion on finitely dimensional spaces the ideas were to use different topologies on *E*, and then the study has taken two directions. One direction is to impose strong topology assumptions which can be found in different works, for example, [8–12]. The other approach is to utilize weak topology assumptions; it is observed that if the Banach space *E* is reflexive we recover locally compactness by endowing it with the weak topology. In [9, 13–15] the Cauchy problem (1) has been discussed in reflexive Banach space. Astala [16] proved that a Banach space *E* is reflexive if and only if (1) admits a local solution for every weakly continuous map *f*. Thus there is no hope to extend Peano's theorem in the weak topology setting to nonreflexive spaces. The nonreflexive case was examined by, among others, [17–20] on assuming, besides the weak continuity of *f*, some condition on *f* involving the measure of weak noncompactness to, somehow, recover the locally compactness lost by the fact that the Banach space we are working on is no longer reflexive. There are a lot of works devoted to investigating uniqueness criteria in which Kamke's original hypothesis is replaced by a dissipative-type condition formulated in terms of a semi-inner product [8, 11, 20–28]. Majorana [29] found out a very close relation between an auxiliary scalar equation ASE of the form

(2) $$\begin{array}{c} u=tf\left(t,u\right) \end{array}$$

and the classical Cauchy problem (1), where *x* and 0 are reals.

**Theorem 2 (see [29])**

Let the function *f*(*t*, *x*) be defined in [0, *a*] × ℝ and continuous with respect to *t* such that *f*(*t*, 0) = 0 for every *t* ∈ [0, *a*]. Further let Cauchy problem (1) have two different classical solutions defined in *t* ∈ [0, *α*]. Then, for every *ε* > 0, there exists *t* ∈ (0, *α*] such that (2) has at least two different roots *u* with |*u* | <*ε*.

An immediate consequence of this latter theorem is the following uniqueness criterion.

**Theorem 3 (see [29])**

Let the function *f*(*t*, *x*) be defined in [0, *a*] × ℝ and continuous with respect to *t*, such that *f*(*t*, 0) = 0 for every *t* ∈ [0, *a*]. Further let there exist *ε* > 0 such that *u* = 0 is the only root of (2) with |*u* | <*ε* for every *t* ∈ [0, *α*]. Then, for (1), *x*(*t*) = 0 is the only classical solution defined in *t* ∈ [0, *α*].

Therefore we have, in ℝ, a very close relation between (2) and (1). It is one of the goals of this work to retain this relation in a suitable generalized sense. However, Majorana's results are not directly extendable to an arbitrary abstract space as the following example shows.

**Example 4**

Let us consider the Cauchy problem:

(3) $$\begin{array}{c} x^{\prime }=f\left(t,x\right),\;\;x\left(0\right)=0, \end{array}$$

where

(4) f(t,x) ={(2||x||(x1+x2),2||x||(x2−x1)),if  x≠0,0,if  x=0.

In polar coordinates (3) becomes

(5) $$\begin{array}{c} r^{\prime }=2\sqrt{r},\;\;\theta^{\prime }=\frac{-2}{\sqrt{r}}. \end{array}$$

Thus, besides the trivial solution *x* = 0, there is at least one other given by

(6) $$\begin{array}{c} x=\{\begin{array}{cc} \left(t^{2}\cos\;\ln\frac{1}{t^{2}},t^{2}\sin\;\ln\frac{1}{t^{2}}\right), & \text{if}\;t\neq 0,\\ 0, & \text{if}\;t=0. \end{array} \end{array}$$

We conclude that (3) satisfies the assumptions of Theorem 10; however, one cannot find more than the trivial root to the auxiliary scalar equation ASE corresponding to (3); namely,

(7) $$\begin{array}{c} u_{1}=\lambda \left(u_{1}+u_{2}\right),\;\;u_{2}=\lambda \left(u_{2}-u_{1}\right), \end{array}$$

where *λ* is an arbitrary scalar.

In his paper [30] the author provided a generalization of Majorana's theorem in finite dimensional Hilbert spaces; a natural question arises: can we extend this result to infinitely dimensional Banach spaces? The answer is positive as will be shown in the following.

A way to provide a version of Majorana's uniqueness theorem in Banach space consists in replacing (2) with a suitable one. Our main concern in this work is the classical Cauchy problem (1), where *f* takes values in a real Banach space *E* and *x* and 0 are in *E*.

Before starting the main work we will introduce some concepts. Throughout the following ||·|| stands for the norm in *E*. The underlying idea to provide counterpart to (2) is based on the following definition.

**Definition 5 (see [11])**

Let *E* be a real Banach space. A subset *P* of *E* is called a cone if the following are true:

- *P* is nonempty and nontrivial (i.e., *P* contains a nonzero point);
- *λP* ⊂ *P*, *λ* > 0;
- *P* + *P* ⊂ *P*;
- $\bar{P}=P$, where $\bar{P}$ denotes the closure of *E*;
- *P*∩−*P* = 0, where 0 denotes the zero element of the Banach space *E*.

Assume that *P* ^0^ ≠ *ϕ*, *P* ^0^ denotes the interior of *E*. The cone *P* of *E* induces an ordering “≤” by setting

(8) $$\begin{array}{c} x\leq y⟺y-x\in P,\\ x<y⟺y-x\in P^{0}. \end{array}$$

Let P* be the set of all continuous linear functionals *c* on *E* such that *c*(*x*) ≥ 0 for all *x* ∈ *P* and let *P*_0_* be the set of all continuous linear functionals *c* on *E* such that *c*(*x*) > 0 for all *x* ∈ *P* ^0^. The underlying idea to provide counterpart to (2) is based on the following Lemma which is due to Mazur [31].

**Lemma 6 (see [11])**

Let *P* be a cone with nonempty interior *P* ^0^; then the following hold:

- *x* ∈ *P* is equivalent to *c*(*x*) ≥ 0 for all *c* ∈ P*;
- *x* ∈ ∂*P* implies that there exists a *c* ∈ *P*_0_* such that *c*(*x*) = 0, where ∂*P* denotes the boundary of *P*.

It is well known that the requirements on the function *f* are dependent on types of solutions; since we concentrate ourselves to weak solutions, then the classical case in the subject is that due to Szép [15]. Before giving Szép's Theorem we need the following definition.

**Definition 7 (see [11])**

A function *f*(*t*, *x*) is said to be weakly-weakly continuous at (*s*, *y*) if given *ε* > 0 and *φ* ∈ E* there exist *δ*(*ε*, *φ*) > 0 and *ℷ*(*ε*, *φ*) a weakly open set containing *y* such that |*φ*(*f*(*t*, *x*) − *f*(*s*, *y*))|<*ε* whenever |*t* − *s* | <*δ* and *x* ∈ *ℷ*.

**Definition 8**

A function *x*(*t*) is said to be weakly differentiable at *t*_0_ if there exists a point in *E* denoted by *x*′(*t*_0_) such that *φ*(*x*′(*t*_0_)) = (*φx*)′(*t*_0_) for every *φ* ∈ E*.

**Theorem 9 (see [15])**

Let *E* be a reflexive Banach space and let *f* be a weakly-weakly continuous function on *A* : 0 ≤ *t* ≤ *a*, ||*x*|| ≤ *b*. Let ||*f*(*t*, *x*)|| ≤ *M* on *A*. Then (1) has at least one weak solution defined on [0, *α*], *α* = min⁡{*a*, (*b*/*M*)}.

## 2. Main Result

This section contains the main results. Throughout this section we will assume that *E* is a real reflexive Banach space endowed with weak topology. *P* ⊂ *E* is a cone with nonempty interior.

**Theorem 10**

Let *f* : [0,1] × *E* → *E* be a weakly-weakly continuous such that ||*f*(*t*, *x*)|| ≤ *M* on [0,1] × *E* and let *f*(*t*, 0) = 0 for every *t* ∈ [0,1]. Suppose further that (1) admits two different weak solutions defined in [0, *α*]  (*α* < *a*). Then, for every *ε* > 0 and every *c* ∈ *P*_0_*, there exists *t* ∈ [0, *α*] such that the following scalar equation

(9) $$\begin{array}{c} c\left(u\right)=tc\left(f\left(t,u\right)\right) \end{array}$$

has at least two different roots *u* with ||*u*|| < *ε*.

Science *E* is is open, there exist *a*, *b* > 0 such that all points whose distance from (0,0) ∈ [0,1] × *E* satisfies 0 ≤ *t* < *α* and  ||*x*|| < *β* are contained in [0,1] × *E*. Let *A* : 0 ≤ *t* ≤ *a* and  ||*x*|| ≤ *b* be such that *A*⊆0 ≤ *t* < *α* and  ||*x*|| < *β*. Then Theorem 9 applied to (1) guarantees the existence of the solution of (1) on [0,1] × *E*. The idea of the proof comes from [29].

**Proof**

It follows, by the assumption *f*(*t*, 0) = 0, that (1) has the zero solution, so we assume that (1) has the weak solution *y*(*t*) ≠ 0.

Let *ε* > 0 and *c* ∈ *P*_0_* be given. Since *u* = 0 is a root of (9) for every *t* ∈ [0, *a*], it is sufficient to show that there exists *t* ∈ [0, *α*] for which (9) is satisfied by some *u* ≠ 0 with ||*u*|| < *ε*. Let a real-valued function *A*_*c*_(*t*) be defined by setting

(10) $$\begin{array}{c} A_{c}\left(t\right)=\{\begin{array}{cc} \frac{c\left(y\right)}{t}, & t\neq 0,\\ 0, & t=0, \end{array} \end{array}$$

*t* ∈ [0, *a*]. Of course *A*_*c*_(*t*) is nonnegative weak continuous in [0, *a*] and weakly differentiable in (0, *a*), and for every *t* in (0, *a*) we have

(11) $$\begin{array}{c} A_{c}^{\prime }\left(t\right)=\frac{1}{t^{2}}\left[tc\left(f\left(t,y\left(t\right)\right)\right)-c\left(y\right)\right]. \end{array}$$

Now, fix *t*_2_ ∈ (0, *a*) with *y*(*t*_2_) ≠ 0 such that ||*y*(*t*)|| < *ε* for every *t* ∈ [0, *t*_2_]. Denote *t*_1_ = sup⁡{*t* ∈ [0, *t*_2_] : *A*_*c*_(*t*) = 0}. Clearly *y*(*t*_1_) = 0 and *y*(*t*) ≠ 0 for every *t* ∈ (*t*_1_, *t*_2_]. At this point there are just two possibilities.

P1: If there exists a *t* ∈ (*t*_1_, *t*_2_] such that *A*_*c*_′(*t*) = 0, then, from (11) for such a *t*, (9) is satisfied by *u* = *y*(*t*). Hence the proof is accomplished by just taking these *t* and *u* = *y*(*t*).
P2: Otherwise if *A*_*c*_′(*t*) ≠ 0 for every *t* ∈ (*t*_1_, *t*_2_]. According to Darboux property *A*_*c*_′(*t*) has a constant sign in (*t*_1_, *t*_2_], then we take *u* = *y*(*t*_2_)(≠0) and define
  (12) $$\begin{array}{c} G\left(t\right)=tc\left(f\left(t,y\left(t_{2}\right)\right)\right)-c\left(y\left(t_{2}\right)\right),\;\text{for}\;\text{every}\;t\in \left[0,a\right]. \end{array}$$

Now let us suppose that *A*_*c*_′(*t*) > 0 for every *t* ∈ (*t*_1_, *t*_2_]. *G*(0) = −*c*(*y*(*t*_2_)) < 0. On the other hand, we have *G*(*t*_2_) = *t*_2_^2^ *A*_*c*_′(*t*_2_) > 0. It follows, by continuity of *G*, that there exists a *t* ∈ (0, *t*_2_] such that *G*(*t*) = 0. The fact that *A*_*c*_(*t*) > 0 for every *t* ∈ (*t*_1_, *t*_2_] and *A*_*c*_(0) = 0 implies that *A*_*c*_′(*t*) < 0 is impossible and the proof will thus be accomplished.

An immediate consequence of Theorem 2 is the following uniqueness criterion.

**Theorem 11**

Let the hypotheses of the Theorem 10 hold and let *f*(*t*, 0) = 0 for every *t* ∈ [0,1]. Assume further that there exist *ε* > 0, *c* ∈ *P*_0_*, and *t*_0_ ∈ (0,1] such that *u* = 0 is the only root of the auxiliary scalar equation (9) with ||*u*|| < *ε* for every *t* ∈ [0, *t*_0_]. Then (1) admits in the interval [0, *t*_0_] only the zero solution.

As it was pointed out by Majorana the crucial point in Theorems 10 and 11 is the assumption that (1) has the zero solution. One follows Majorana's procedure to remove this restriction. If we know a weak solution *y* of (1), then, by means of change of variables *x* = *p* + *y*(*t*), (1) becomes

(13) $$\begin{array}{c} p^{\prime }=F\left(t,p\right),\;\;p\left(0\right)=0, \end{array}$$

where *F*(*t*, *p*) = *f*(*t*, *p* + *y*(*t*)) − *f*(*t*, *y*(*t*)). It is clear that any two different weak solutions of (1) are mapped to different weak solutions of (13). Moreover (13) admits the zero solution 0, which corresponds to the solution *y* of (1). One thus gets the counterpart to (9):

(14) $$\begin{array}{c} c\left(u\right)=tc\left(F\left(t,u\right)\right). \end{array}$$

We can restate Theorems 10 and 11 involving the nontrivial solution *y* instead of *x* = 0.

**Theorem 12**

Let *f* : [0,1] × *E* → *E* be a weakly-weakly continuous such that ||*f*(*t*, *x*)|| ≤ *M* on [0,1] × *E* and let *y* be a weak solution of (1). Further let (1) admit two different weak solutions defined in [0, *α*]  (*α* < *a*). Then, for every *ε* > 0 and for every *c* ∈ *P*_0_*, there exists *t* ∈ [0, *α*] such that (14) has at least two different roots *u* with ||*u*|| < *ε*.

**Theorem 13**

Let *f* : [0,1] × *E* → *E* be a weakly-weakly continuous such that ||*f*(*t*, *x*)|| ≤ *M* on [0,1] × *E* and let *y* be a weak solution of (1). Assume further that there exist *ε* > 0, *c* ∈ *P*_0_*, and *t*_0_ ∈ (0,1] such that *u* = 0 is the only root of the auxiliary scalar equation (14) with ||*u*|| < *ε* for every *t* ∈ [0, *t*_0_]. Then the (1) admits in the interval [0, *t*_0_] only the weak solution *y*.

**Remark 14**

Another critical point is that, as far as we know, in the standard reflexive Banach spaces usually encountered in differential equations, that is, *L* ^*p*^(*Ω*) spaces with *Ω* being a measurable subset in ℝ^*n*^ and *p* ∈ (1, *∞*), the corresponding positive cones have empty interior. Except for the finite dimensional space, the only nontrivial example of a positive cone with nonempty interior is in *C*(*K*), where *K* is a compact topological space. But *C*(*K*) is not reflexive. This motivates us to give the following example.

**Example 15**

We think, Sobolev spaces *H* ^1^ and *H* ^2^ are the required examples. Because they are Hilbert spaces. *H* ^1^ consists of continuous functions and *H* ^2^ consists entirely of continuously differentiable functions. See page 382 of [32].

## 3. Conclusion

We have taken the time interval to be [0; 1] and the initial value *x*(0) to be 0 only for simplicity of notation; our argument would work just as well for any other compact interval and any other initial value. Moreover, Szép's assumptions can be replaced by any set of sufficient conditions that guarantee existence of solutions for (1) in reflexive as well as in nonreflexive Banach spaces.
